# Quincy Medical Society

**Published:** 1867-01

**Authors:** Addison Niles

**Affiliations:** Sec’y.


### QUINCY MEDICAL SOCIETY.

---

The semi-annual meeting of the Quincy Medical Society was held at the office of Dr. Zimmermann, in Quincy, Nov. 18th, 1866.

A quorum being present, the President, Dr. C. A. W. Zimmermann, called the meeting to order. The proceedings of the annual meeting having been read and approved, the President proceeded to deliver his able and elaborate essay upon Bright's Disease. The manner in which he treated the subject may be inferred from the following extract from the exordium:—

"I discharge this obligation with no inconsiderable degree of pleasure, inasmuch as this subject is classed among the most interesting in pathology; and I intend discussing it by strictly following the medical literature as far as it deals with facts, and particularly as far as it touches the anatomy of this disease, and eschews hypothesis; while I shall sometimes mention my own experience, which is naturally enough based on observations in view of the sick couch, and the therapeutics of the disease, which are without exception open for discussion." The President entered at length into the consideration of the causes, pathology, diagnosis, prognosis, and treatment of Bright's disease.

On motion, resolved, that the thanks of this Society be tendered to the President for his able essay, and that a copy is hereby requested for publication.

The Secretary then read the history and treatment of two cases which had come under his observation since the annual meeting:—

Case I. *June 21st, 1866.* Was requested to visit Mrs. S., aged 25 years. I learned from her that she had been confined to the bed seven weeks, and had vomited, most of the time, every hour. The matter ejected is described as green and yellow; urine natural in quantity, specific gravity 1.011, acid, not albuminous. The last menstrual period occurred about four months since, as near as she recollects. She is greatly emaciated, very feeble, and takes but little nourishment. The mamma is flaccid and wasted, no change has occurred in the nipple or in the areola about it. On vaginal examination, the uterus was found enlarged, and the os hard and fissured. Prescribed one teaspoonful of Ellis' wine of pepsin in a wineglass of water three times a-day, and one tablespoonful of lime-water and two of milk every two hours, with beef-tea for nourishment.

*22d.* The symptoms not materially changed. Directed the treatment to be continued.

*23d.* Patient no better; has vomited every hour, and has not slept during the night. A consultation was agreed upon, and Dr. L. H. Baker of this city called. After a careful examination, it was decided that there is no evidence of a living fœtus in the uterus, but even if there were, the dangerous condition of the mother demands interference. A speculum was introduced and the *os* brought to view, and a sound passed into the uterus three inches, no fluid followed its withdrawal. A sponge tent was then passed into the *os* and the patient directed to take the medicine as before.

*24th.* The patient has not vomited as much as formerly, and has taken more nourishment. The tent is not to be found in the vagina, and cannot be seen in the os through the speculum, and the supposition is entertained that it may have passed away unobserved. Introduced a larger tent, and directed treatment to be continued.

*25th.* The vomiting ceased during the day and returned at night. On examination, the tent cannot be found; introduced another with a small cord attached to it.

*26th, 9 o'clock A.M.* Has vomited less than formerly. Removed the tent and introduced another. *2 o'clock P.M.* Quite an offensive discharge has occurred, and the patient complains of pain in the back. Removed the tent and found the *os* considerably dilated; on passing the finger, the two tents first used were found within the *os* and removed.

*27.* The patient is more quiet and vomits less. Continue the medicine.

*28.* The remains of a fœtus passed away during the night; it is six inches in length, and weighs one ounce troy; it is much wasted; the skin is wanting; the head collapsed; the walls of the chest and abdomen wanting; no trace is found of the lungs, diaphragm, stomach, or intestines; the heart, liver, and kidneys were observed in their natural positions. The fœtus has, doubtless, been dead several weeks, and the placenta probably been absorbed, as it has not passed away, and cannot be discovered on examination. No flooding has occurred. Prescribed 5 grs. of pulvis ergot, to be taken every three hours, and a teaspoonful of a saturated solution of sulphite of soda three times a-day.

*30th.* The vomiting continues frequently, and is supposed to be excited by the medicine, which was discontinued, and the wine of pepsin and lime-water resumed, and milk freely taken for nourishment.

*July 1st.* The vomiting has now ceased, and the patient's condition satisfactory in every respect. No unfavorable symptoms afterwards occurred, and the patient made a very rapid recovery.

*Remarks.*—The irritation of a dead fœtus in the uterus has been referred to by authors as one of the causes of abortion; but it is evident that it does not always give rise to this result. Tyler Smith refers to a case, in which the fœtus died at the fourth month, but was not expelled until the full term. As the sound of the fœtal heart cannot ordinarily be heard until after the fourth month, we have no certain evidence by which we can infer the death of the fœtus. In the cases which have come under my observation, I have found the mamma flaccid, the nipple and its areola without any of the ordinary indications of pregnancy, the neck of the uterus harder, and the os more patulous than usual in that condition. It seems desirable that more careful and extended observations be made, as to the symptoms which indicate the presence of a blighted fœtus *in utero*. In this case, if its presence had been known at an earlier period, the patient might have been saved several weeks of distressing illness.

Case II. Was called to visit Mr. B., aged 35, in consultation with Dr. L. H. Baker, of this city, September 3d, 1866, and found the patient suffering from narcotism. The following is the history of the case, as given by the attendants:—

The attention of his wife was called to him by an unusual snoring, and, on going into his room, she found him quite insensible, and nearly black in the face and neck. Supposing him to be in a fit, she attempted to give him some wine, spirits of camphor, and other restoratives, of which he swallowed but little, and that at the risk of strangling. Just at that time, she found in his pocket an empty bottle, labelled laudanum.

Dr. Baker was called immediately, and gave him, 10 minutes before 4 o'clock, 20 grs. of sulphate of zinc, and repeated the dose in 5 minutes, without effect. I arrived 25 minutes after the emetic had been given. Not having had its effect, it was thought best to use the stomach pump. About one quart of warm water was thrown by it into the stomach and immediately pumped out with the whole of its contents, which gave a strong odor of laudanum. This was repeated three times, and about a gallon of water, in all, was used, the last of which was returned nearly clean and devoid of smell of laudanum. During this time, the patient remained entirely insensible, with the eyes closed, the pupils contracted, respiration slow and stertorous, pulse slow, with other symptoms of narcotism.

After thoroughly emptying the stomach, a galvanic battery was used for nearly two hours, one pole applied to the upper part of the spine and the other to the front of the chest. About one hour after the stomach was evacuated, and while using the battery, the patient began to sink, the respiration became reduced to four in a minute. Dr. Baker resorted to artificial respiration (in addition to the battery) which was kept up by by Hall's method, about a-quarter of an hour, until an improvement occurred in the respiration, which continued until the patient was fully restored. No unpleasant symptoms followed excepting vomiting, which occurred at intervals during the night.

It was subsequently ascertained that the patient took two ounces of tincture of opium at 10 o'clock A.M., and also two ounces at 1 o'clock P.M; and that he was not in the habit of taking opium or laudanum; that he took it for a diarrhœa, which he had for three or four days. The apothecary says the tincture of opium sold the patient contained just one-half the officinal quantity of opium.

On motion, Dr. J. F. McCormic was appointed essayist for the next meeting.

On motion, Dr. A. Niles was appointed delegate to the State Society.

On motion, resolved, that the proceedings of this meeting be published in the CHICAGO MEDICAL EXAMINER, and an abstract of them in our city papers.

ADDISON NILES, *Sec'y.*

